# *Physalis angulata* Leaf Extract Attenuates H_2_O_2_-Induced Neurotoxicity in Zebrafish Through Metabolomic Evidence of Antioxidant Pathway Restoration

**DOI:** 10.3390/antiox15060758

**Published:** 2026-06-16

**Authors:** Fanny Anabel Sulistio, Richard Johari James, Salfarina Ramli, Callista Andinie Mulyadi, Hasseri Halim, Michael Vin, Fadlina Chany Saputri, Indri Yuliani Hamdani, Syariful Mubarok, Aliyya Hanaa Naila, Amorisa Savitri, Donna Maretta Ariestanti

**Affiliations:** 1 Faculty of Pharmacy, Universitas Indonesia, Depok 16424, Indonesia; fanny.anabel@ui.ac.id (F.A.S.); mulyadi.callista@farmasi.ui.ac.id (C.A.M.); michael.vin@ui.ac.id (M.V.); fadlina.chany@farmasi.ui.ac.id (F.C.S.); indriyuliani28@farmasi.ui.ac.id (I.Y.H.); aliyya.hanaa51@ui.ac.id (A.H.N.); amorisa.savitri@ui.ac.id (A.S.); 2 Integrative Pharmacogenomics Institute (iPROMISE), Universiti Teknologi MARA, Puncak Alam 42300, Malaysia; richard@uitm.edu.my (R.J.J.); salfarina2892@uitm.edu.my (S.R.); hasseri2945@uitm.edu.my (H.H.); 3 Faculty of Pharmacy, Universiti Teknologi MARA, Puncak Alam 42300, Malaysia; 4 Department of Agronomy, Faculty of Agriculture, Universitas Padjadjaran, Bandung 45363, Indonesia; syariful.mubarok@unpad.ac.id

**Keywords:** physalis angulata, antioxidant, neuroprotective, metabolomic, zebrafish

## Abstract

Neurological disorders affect billions of people worldwide, with oxidative stress and its associated cellular mechanisms recognized as key pathological drivers of neuronal damage. Despite this, natural agents capable of targeting these mechanisms remain underexplored. *Physalis angulata* L. (PA) contains physalins and withanolides known for their neuroprotective properties, though its in vivo neuroprotective mechanism remains poorly characterized. This study evaluated the preventive neuroprotective effects of PA leaf extract using an integrated developmental, behavioral, and metabolomic approach in a H_2_O_2_-induced zebrafish (*Danio rerio*) model. PA extract demonstrated moderate-to-strong antioxidant capacity (ABTS IC_50_ = 66.66 ppm; DPPH IC_50_ = 114.91 ppm). Treatment with 50 ppm PA extract provided optimal neuroprotection, significantly restoring body length, yolk sac utilization, and heart rate, while markedly improving locomotor activity and reducing anxiety-like thigmotaxis behavior. Notably, PA extract demonstrated superior efficacy compared to N-acetylcysteine (NAC) across multiple developmental and behavioral endpoints. Metabolomic profiling of zebrafish embryo homogenates provided direct in vivo biochemical evidence of antioxidant pathway modulation, demonstrating that PA extract mitigated metabolic disruption by restoring amino acid metabolism (glycine, serine, and threonine), glutathione synthesis, glycerophospholipid homeostasis, and one-carbon pool metabolism. These findings demonstrate that *P. angulata* exerts multi-target neuroprotection by restoring antioxidant pathways and promoting metabolic homeostasis, supporting its potential as a natural therapeutic candidate for oxidative stress-related neurological conditions.

## 1. Introduction

Neurological disorders represent a major global health burden, encompassing neurodegenerative diseases and neurodevelopmental disorders across all age groups [1,2,3]. Among the overlapping pathophysiological mechanisms underlying these conditions, oxidative stress plays a central role by triggering neuronal damage through mitochondrial dysfunction, impaired synaptic signalling, and neuroinflammation [4,5,6]. Despite the escalating global burden, conventional pharmacotherapy offers limited symptomatic benefits with notable side effects [7,8,9], underscoring the need for alternative neuroprotective agents that address underlying mechanisms.

Natural products have gained increasing attention as potential neuroprotective agents due to their diverse bioactive constituents and multi-target mechanisms of action [10,11]. Among these, *Physalis angulata* has emerged as a compelling candidate. *P. angulata* is a medicinal plant known for its rich content of physalins and withanolides [12,13,14], compounds reported to possess strong antioxidant potential, promote synaptic reconstruction during axonal and dendritic regeneration, and confer neuronal protection. In addition to withanolides, *P. angulata* is characterized by a diverse phytochemical profile encompassing flavonoids, terpenes, carotenoids, alkaloids, saponins, tannins, glycosides, and phenolic compounds [15,16,17]. These constituents collectively contribute to significant biological activities, including anti-inflammatory effects, high antioxidant capacity, immunomodulatory activity, inhibition of oxidative stress, and inhibitory activity against acetylcholinesterase (AChE) and butyrylcholinesterase (BChE) [15,17,18,19,20]. However, the safety profile of *P. angulata* requires further investigation due to reports of hepatotoxic effects associated with withanolide-containing compounds [21,22].

Despite the well-documented phytochemical properties of *P. angulata*, its neuroprotective effects at the developmental, behavioral, and metabolomic levels in a single in vivo model have not been comprehensively evaluated [23]. The mechanistic basis linking its phytochemical constituents to observed biological effects remains insufficiently characterized, representing a critical gap in the literature.

Therefore, this study aimed to evaluate the neuroprotective effects of *P. angulata* leaf extract administered prior to oxidative stress induction, through an integrated approach combining phytochemical characterization, in vivo developmental and behavioral assessments, and untargeted metabolomic profiling in a H_2_O_2_-induced zebrafish (*Danio rerio*) model. The zebrafish model was selected for its well-established utility in neurotoxicology research, high reproductive capacity, morphological and physiological similarities to mammals, optical transparency for in vivo observation, and conserved neurotransmitter systems relevant to neurobehavioral studies [24,25,26]. To our knowledge, this is the first study to employ an integrated three-tier approach, combining developmental toxicity assessment, locomotor behavioral analysis, and untargeted metabolomic profiling, to characterize the neuroprotective mechanism of *P. angulata* ethanolic leaf extract in a zebrafish model of H_2_O_2_-induced oxidative stress.

## 2. Materials and Methods

### 2.1. Plant Material and Extract Preparation

Dried leaves of *P. angulata* were obtained from the Pusat Studi Biofarmaka Tropika, LRI-PGK IPB, Bogor, West Java, Indonesia. The plant material was authenticated at the same institution, and a voucher specimen was deposited under the reference number BMK0094082016. Leaves were selected due to their higher total phenolic and flavonoid contents relative to the fruit [27]. The dried leaf powder was then extracted using Ultrasonic-Assisted Extraction (UAE) with 96% ethanol for 1 h. The dried powder-to-solvent ratio was 1:10, and the extraction was performed at 25 ± 5 °C. The extraction process included one re-extraction step, resulting in a total of two extraction cycles per batch. The resulting extract was filtered and concentrated using a rotary evaporator to yield the final extract (Figure S1).

### 2.2. Total Flavonoid Content (TFC)

TFC was determined using the colorimetric method described previously [25]. Quercetin was used as the standard for calibration (70 to 240 µg/mL concentration). A volume of 500 µL of each standard or sample was mixed with 1.5 mL ethanol. Subsequently, 100 µL of 10% aluminum chloride was added, followed by 100 µL of sodium acetate 1 M and 2.8 mL of distilled water. The mixture was incubated for 30 min at room temperature. The experiment was performed in triplicate, and the absorbance was measured at 431 nm. TFC was expressed as mg quercetin equivalent (QE)/g extract.

### 2.3. Total Phenolic Content (TPC)

TPC of the extract was measured using the Folin–Ciocalteu (FC) method. Gallic acid (GAE) was used as a standard for calibration (150 to 400 µg/mL concentration). One milliliter of each standard or sample was mixed with 5 mL of 7.5% (*v*/*v*) FC reagent (Merck, Darmstadt, Germany) and preincubated for 8 min, followed by addition of 4 mL of 1% sodium hydroxide solution. The mixture was incubated for one hour at room temperature, and the absorbance was measured at 710 nm. The experiment was conducted in triplicate. Results were expressed as mg gallic acid equivalent (GAE)/g extract.

### 2.4. Metabolomic Profiling of P. angulata Extract

Metabolomic profiling of the extract was conducted to identify the constituent compounds of the extract. The analysis was performed using 6520 LC-MS/QTOF (Agilent Technologies, Santa Clara, CA, USA) with a ZORBAX Eclipse Plus C18 column (100 × 2.1 mm, 1.8 µm; Agilent Technologies, Santa Clara, CA, USA), a flow rate of 0.25 mL/min, and 0.1% formic acid as the mobile phase over 30 min. Detection was performed in full scan mode using positive ESI. Data was processed with Agilent MassHunter Qualitative Software (Version B.03.01), and compound identification was based on comparison of mass spectra with the METLIN database. For analysis, ten milligrams of sample was dissolved in LC-grade ethanol, centrifuged at 10,000 rpm for 10 min, and filtered using a nylon filter before injection into the LC-MS Q-TOF system.

### 2.5. Antioxidant Assay

The antioxidant activity of PA extract used in this study was measured using DPPH (2,2-diphenyl-1-picrylhydrazyl) & ABTS (2,2′-azino-bis(3-ethylbenzothiazoline-6-sulfonic acid)) scavenging assay, as well as through FRAP (Ferric Reducing Antioxidant Power) measurement. The ABTS and DPPH scavenging assays were performed following a previously established method, with modifications to achieve optimal absorbance readings within the 0.2–0.8 range [26]. To measure extract-scavenging ability, the ABTS reagent was prepared by mixing 5 mL of 7 mM ABTS solution with 88 µL of 140 mM potassium persulfate. After 16 h of incubation, the reagent was diluted with distilled water at a 1:30 ratio. For the sample, 100 µL PA extract (25, 50, 100, 150, 200, and 250 ppm) was pipetted into a 96-well microplate and then diluted with 100 µL of ABTS. Absorbance was measured using a microplate reader at 735 nm. Ascorbic acid (1, 3, 4, 5, 6, and 8 ppm) was included as a positive control, and its IC_50_ was calculated for comparison. Methanol was used as a control blank, and a methanol-ABTS reagent mixture as a sample control.

Subsequently, DPPH reagent was prepared by diluting DPPH powder to a final concentration of 100 ppm. For sample measurement, 100 µL of PA extract (50, 100, 160, 240, 300, and 360 ppm) was pipetted into a 96-well microplate and mixed with an equal volume of DPPH. After 30 min of incubation at room temperature, absorbance was measured using a microplate reader at 514 nm. Ascorbic acid was assayed in parallel as a positive control at concentrations of 3, 5, 7, 9, 12, and 14 ppm, and its IC_50_ was calculated for comparison. As in the ABTS measurement, a mixture of 200 µL p.a. methanol was used as a control blank, and a methanol–DPPH reagent mixture (1:1) as a sample control. The antioxidant activities measured by ABTS and DPPH assays are reported as IC_50_ values.

Additionally, Ferric-Reducing Antioxidant Power (FRAP) was measured using a previously described method [28], with modifications. For the calibration curve measurement, the FRAP reagent was prepared by mixing 300 mM acetate buffer, 10 mM TPTZ (2,4,6-tripyridyl-s-triazine), and distilled water in a 10:1:1 (*v*/*v*/*v*) ratio. Concurrently, the FRAP reagent for sample and ascorbic acid standard measurements was prepared by mixing 300 mM acetate buffer, 20 mM FeCl_3_·6H_2_O, and 10 mM TPTZ (10:1:1, *v*/*v*/*v*). Standard curve measurements were performed by diluting FeSO_4_ in distilled water to obtain concentrations of 10, 15, 25, 30, 40, and 50 µM. For sample measurement, the extract was diluted to a final concentration of 300 ppm. Additionally, 10 ppm ascorbic acid was prepared as a standard. Then 50 µL of each concentration and mixture was pipetted into a 96-well microplate and mixed with 150 µL of the corresponding FRAP reagent. Plates were shaken for 10 s and incubated for 30 min. The absorbance was measured using a microplate reader at 594 nm. FRAP results are expressed as g FeSO_4_·7H_2_O equivalents per 100 g of sample and as AEAC (ascorbic acid equivalent antioxidant capacity) values.

### 2.6. Animal Housing and Husbandry

AB strain adult zebrafish were housed in an automated aquatic system (ZebTEC Active Blue Stand Alone, Tecniplast, Buguggiate, Italy). The system was set to mimic natural habitat conditions of water flow, temperature (26 ± 1 °C), and pH (7.0 ± 0.5). Water pH was maintained using sodium bicarbonate, while conductivity was adjusted using sea salt (Instant Ocean^®^, Spectrum Brands, Middleton, WI, USA). Breeding was performed using sexually mature zebrafish (at least 3 months old). After spawning, the broodstock were removed, and the eggs were transferred to an egg-water solution containing a diluted salt solution at 60 μg/mL. The collected embryos were transferred to a 24-well culture plate, and treatments were conducted at 26 ± 1 °C. The zebrafish larvae used in the study were tested up to 120 h post-fertilization (hpf) and thus were not subject to ethical regulations in accordance with the European Union Directive 2010/63/EU [29,30].

### 2.7. Experimental Groups and Exposure Design

A total of 240 fertilized embryos were randomly assigned to six treatment groups, with 40 embryos per group. The six treatment groups used in the research were as follows: a control group consisting of embryos exposed to only egg water; an H_2_O_2_ group consisting of embryos only treated with 0.5 mM H_2_O_2_; a N-acetylcysteine (NAC) group as positive control consisting of embryos treated with NAC (5 μM, a concentration selected based on a previous study [31]); and PA extract-treated embryos at concentrations of 12.5, 25, and 50 ppm. Embryos were distributed into 24-well plates, with 5 embryos per well. The concentration was chosen based on the previous study. For the NAC- and PA-treated groups, pre-treatment was performed by adding 2 mL of the respective treatment solution to each well, followed by incubation for 2 h at 26 °C. This pre-treatment design was employed to evaluate the preventive neuroprotective potential of PA extract, rather than its therapeutic effect. After pre-treatment, 9 μL of 0.1163 M H_2_O_2_ stock solution was added to each well, thus achieving a 0.5 mM H_2_O_2_ concentration. In the control and H_2_O_2_ groups, 2 mL of egg water was added; only the H_2_O_2_ group subsequently received the H_2_O_2_ stock solution. Throughout the treatment period, embryos and plates were maintained in an incubator at 26 ± 1 °C. The treatment solution was replaced daily (Figure S2).

### 2.8. Developmental Toxicity Assays

The developmental toxicity assay was conducted following an established protocol [32], with modifications. Several parameters were monitored to assess the developmental status of each treatment group. First, embryo survival was monitored daily from 48 to 72 hpf. Body length was also measured at multiple developmental stages (72, 96, and 120 hpf) using a stereomicroscope and later analyzed using ImageJ (Version 1.54p) software. The yolk area was also measured using the same technique at 8 and 120 hpf to assess developmental progression. Epiboly progression was qualitatively observed under stereomicroscopy at 6 hpf and reported as a supplementary developmental observation (Figure S5). Malformation including pericardial edema and spinal deformities were recorded at 96 hpf. Lastly, heart rate was recorded at 72 and 96 hpf. Heart rate was quantified by manually counting cardiac beats from 10-s slow-motion video recordings. Each measurement, including epiboly, hatching rate, heart rate, body length, and yolk area, was performed in triplicate.

### 2.9. Behavioral Light–Dark Locomotion Test

The behavioral assay was performed according to a previously described method, with modifications [33]. At 120 hpf, larvae were transferred to fresh well plates and placed individually. The treatment solution was replaced with egg water, and the larvae were allowed to acclimate for 2 h. After incubation, the plates were positioned on a lighting plate, and larval swimming behaviour was recorded under alternating 1-min light and dark cycles for six repetitions. Each measurement was conducted three times. Locomotor activity was recorded and analyzed using ToxTrac (2025 Version 1.1) [34], a visual contrast-based tracking software that requires illumination for organism detection. Quantitative analysis was therefore conducted during light phases only.

### 2.10. Sample Collection and Preparation for Nitric Oxide Measurement

For the nitrite ion (NO^2−^) measurement, 20 zebrafish larvae from each group were collected and transferred into 1.5 mL microcentrifuge tubes. Water was later removed, and each sample was washed using 10× phosphate-buffered saline (PBS). Then, 700 μL of 1× PBS was added to each Eppendorf tube, and larvae were subsequently homogenised using a tissue homogeniser for 60 s. Homogenised samples were then vortexed for 30 s and centrifuged at 3000× *g* for 10 min at 4 °C. Supernatants were removed and used for the nitric oxide assay.

### 2.11. Nitric Oxide Measurement

The nitrite ions (NO_2_^−^) in the zebrafish samples were measured using the Griess assay as previously described [35], with minor modifications. For the Griess reagent, a 1:1 mixture of 1% sulphanilamide and 0.1% N-(1-naphthyl) ethylenediamine dihydrochloride (NED) was prepared. A 1% sulphanilamide solution was made by heating 0.1 g of the reagent in 5 mL of distilled water and 0.25 mL glacial acetic acid, then adjusting the total volume to 10 mL. Separately, a 0.1% NED solution was produced by dissolving 0.01 g of NED in 10 mL of distilled water, which was subsequently diluted to the target concentration of 0.1% (*v*/*v*).

Prior to nitric oxide measurement, the protein concentration of each sample was first determined by the A280 method using the NanoDrop 2000 Spectrophotometer (Thermo Fisher Scientific, Waltham, MA, USA). Protein determination was performed to normalize nitrite levels across samples and to account for differences in larval biomass between groups. For the Griess assay, 150 µL of each sample was mixed with an equal volume of Griess reagent in a 96-well plate and incubated at room temperature for 10 min. Absorbance was then recorded at 540 nm. A standard curve was prepared by dissolving 0.0345 g of NaNO_2_ in PBS to obtain a 1 mM stock solution. The solution was then diluted to 5, 10, 20, 30, 40, 50, and 60 µM. To account for variations in sample concentration, measured nitrite levels (NO_2_^−^) were normalized to total protein content and expressed as μM NO_2_^−^/mg protein.

### 2.12. Developmental Toxicity, Behavioral, and Nitrite Measurement Data Analysis

Results obtained from the developmental toxicity, behavioral, and Griess assays were expressed as the mean ± standard deviation (SD). All statistical analyses were performed using GraphPad Prism version 10.6.1. Data significance was determined using one-way ANOVA followed by Tukey’s multiple comparison test to evaluate pairwise differences between groups. *p*-values < 0.05 were considered statistically significant.

### 2.13. Metabolomics Analysis

Zebrafish larvae were collected at two developmental time points (120 and 96 hpf) for subsequent metabolomic analysis (Figure S3). At 120 hpf, 20 larvae were taken from each group, rinsed with egg water, transferred to Eppendorf tubes, flash-frozen in liquid nitrogen, and stored at −80 °C until extraction. A similar procedure was applied to an independent batch collected at 96 hpf. In this batch, only three treatment groups were included (PA-treated larvae at 12.5, 25, and 50 ppm).

For sample extraction, thawed larvae were homogenized in 1 mL analytical-grade methanol using a Vibra-Cell sonicator (20 kHz, 30 s; Sonics & Materials, Newton, CT, USA). Homogenates were centrifuged (14,000 rpm, 5 min, 4 °C), and the supernatant was filtered through a 0.45 μm syringe filter. The filtrate was concentrated under reduced pressure for approximately 1 h, weighed, and reconstituted in LC-MS-grade methanol to 1 mg/mL. Samples were vortexed (1 min) and centrifuged (14,000 rpm, 10 min, 4 °C) before transferring to LC-MS vials.

Additionally, a pooled sample was prepared by combining 20 μL from each of the 9 samples and used as quality control (QC). A blank sample was also prepared by filling the insert vial with LC-MS grade methanol. All samples were analysed using LC-MS/QTOF (model 6520; Agilent Technologies, Santa Clara, CA, USA). The column used was a ZORBAX Eclipse plus C18 column (100 mm × 2.1 mm × 1.8 μm; Agilent Technologies, Santa Clara, CA, USA) with a temperature set to 40 °C. Mobile phases were 0.1% formic acid in water (A) and 0.1% formic acid in acetonitrile (B), with a gradient from 5% to 95% B over 30 min at 0.25 mL/min (injection volume: 5 μL) in positive ionization mode.

All samples were run in triplicate, with QC samples injected at the beginning, middle, and end of the run. Analytical reproducibility was assessed by calculating the relative standard deviation (RSD) of selected metabolites across QC injections.

### 2.14. Metabolomic Data Analysis

Metabolomic analysis was performed to characterize the biochemical alterations in zebrafish embryos following exposure to oxidative stress (H_2_O_2_) and treatment with the extract and positive control NAC. The LC–MS data sets obtained from each treatment group were subjected to metabolite annotation based on accurate mass and fragmentation pattern matching against major databases, including the Human Metabolome Database (HMDB), PubChem, and KEGG. A total of 44 metabolites were annotated, representing amino acids, purine derivatives, and sphingolipid derivatives. QC samples were used to monitor analytical reproducibility, and their tight clustering confirmed the reliability of the generated data. These annotated metabolites were subsequently analysed through multivariate statistical approaches, namely, Principal Component Analysis (PCA) and Partial Least Squares Discriminant Analysis (PLS-DA), followed by the generation of a hierarchical cluster heatmap to capture treatment-related variations.

## 3. Results

### 3.1. Phytochemical Characterization and Antioxidant Activity of P. angulata Extract

The extraction process produced a crude extract with a yield of 15.64%. The determination of TFC and TPC was carried out on the ethanol extract of *P. angulata* leaves to quantitatively assess flavonoid and phenolic compound levels. Based on the absorbance values obtained, the total flavonoid content in the leaf extract was calculated. The results showed that the average total flavonoid content was 51.06 ± 0.34 mg QE/g extract and the total phenolic content was 59.01 ± 0.79 mg GAE/g extract (Table S1). These values were comparatively higher than those reported by Lestiariani (2023), who applied a similar extraction protocol using 70% ethanol as the solvent, resulting in a flavonoid content of 22.47 mg QE/g extract [36]. In comparison, previous studies reported total phenolic contents of 27.39 and 28.93 mg GAE/g extract, respectively [37,38].

The PA extract used in this study demonstrated moderate to strong antioxidant capacity through multiple mechanisms (Table 1). ABTS and DPPH scavenging assays demonstrated the extract’s ability to scavenge free radicals, while the FRAP assay measured its antioxidant ability by reducing Fe^3+^ ions [26,39]. Specifically, the extract exhibited higher scavenging activity in the ABTS assay (IC_50_ = 66.663 ± 0.72 ppm) compared to the DPPH assay (IC_50_ = 114.913 ± 2.19 ppm). This difference suggests that the bioactive constituents in the PA extract may function more efficiently in a polar-organic medium, such as the ABTS system [27,40]. Furthermore, the FRAP assay results (4.163 ± 0.08 g FeSO_4_ eq/100 g; AEAC of 52.548 ± 0.69 mg AA/g) confirm the extract’s capacity to mitigate oxidative stress through electron donation. In comparison, ascorbic acid standard yielded IC_50_ values of 4.567 ± 0.08 ppm (DPPH) and 2.694 ± 0.05 ppm (ABTS), and a FRAP value of 79.215 ± 0.83 g FeSO_4_ eq/100 g. While PA extract exhibited lower antioxidant potency than ascorbic acid, these findings validate a moderate-to-strong antioxidant profile [41].

When comparing antioxidant results with previous studies, apparent variations among studies are evident. The DPPH value of 114.913 ppm obtained in this research reflects stronger radical scavenging activity than the 190 ± 4.7 ppm reported in previous study. In that same study, the roots and stems exhibited substantially lower antioxidant activity, with markedly higher IC_50_ values of 520 ± 6.0 ppm and 1120 ± 14.5 ppm, respectively [42]. In contrast, another study using methanolic root extract of PA reported substantially lower IC_50_ values for DPPH (58.07 µg/mL) and ABTS (64.37 µg/mL), suggesting that solvent polarity and extraction method can substantially influence antioxidant constituent yield. Furthermore, LC-MS chemical profiling of that root extract revealed a distinct phytochemical composition relative to our study on leaf extract. This suggests that differences in constituent profiles fundamentally influence antioxidant activity between plant parts [43]. Moreover, a multi-part comparative study further reported that the highest antioxidant activity of PA was found in leaves with IC_50_ 158.80 ppm (DPPH) and 165.45 ppm (ABTS). While the same study also reported a substantial FRAP value, direct comparison of FRAP value was limited due to differences in reporting units [44]. Altogether, these variations suggest antioxidant activity is strongly influenced by factors such as botanical source, plant part, extraction solvent, and geographic origin of collection [45]. In the present study, leaf material was harvested from West Java, Indonesia, while other studies used for comparison were harvested from different regions, including East Java [42], Kerala (India) [43], and South Sulawesi [44]. Differences in local growing conditions between collection sites may therefore account for the observed inter-study variability [45]. Despite this inter-study variability, the present study consistently confirms the antioxidant potential of PA extract in agreement with previous literature.

An LC-MS/QTOF analysis was performed on the PA extract. A total of 30 compounds were detected; however, only 14 were successfully annotated based on the METLIN database (Table 2, Figure S4). The identified compounds span several chemical classes, including amino acids, withanolides, flavonoids, chlorophyll derivatives, and fatty acid derivatives.

Among the detected metabolites, 2-amino-3-methyl-1-butanol showed the highest signal intensity (peak height 75,585), followed by D-proline and pheophorbide A, indicating the presence of alcohol amines, amino acids, and chlorophyll derivatives as dominant constituents. Notably, several withanolide compounds, including physalin A, physalin E, and 25,27-dihydro-4,7-didehydro-7-deoxyphysalin A, were identified, highlighting the presence of steroidal lactones of the Solanaceae family. In addition, the detection of robinetin 3-rutinoside, a flavonoid glycoside, and pheophorbide A suggests the contribution of polyphenolic and chlorophyll-derived compounds with known antioxidant properties. Overall, the LC–MS profile demonstrates a chemically diverse extract enriched with withanolides, flavonoids, and chlorophyll derivatives, supporting its potential as a source of bioactive compounds with antioxidant-related activity.

### 3.2. H_2_O_2_-Induced Developmental Toxicity of Zebrafish Embryos

**Figure 1:**
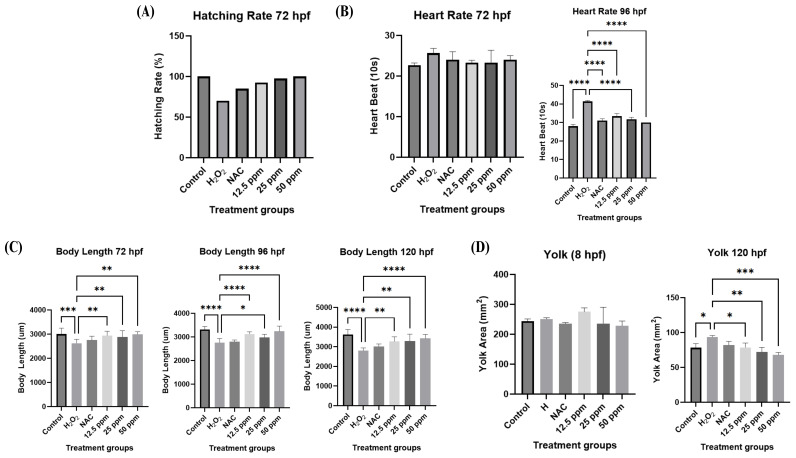
Developmental toxicity measurement results. Embryos were pre-treated with PA extract (12.5, 25, or 50 ppm) or N-acetylcysteine (NAC; 5 μM) prior to H_2_O_2_ (0.5 mM) exposure. (**A**) Hatching rate (*n* = 40 embryos per group). (**B**) Heart rate measured (*n* = 3 embryos per group). (**C**) Body length (*n* = 10 embryos per group). (**D**) Yolk area (*n* = 3 embryos per group). (* = *p*-value < 0.05; ** = *p*-value < 0.01; *** = *p*-value < 0.001; **** = *p*-value < 0.0001).

Epiboly progression data are reported as a supplementary developmental observation (Figure S5). Briefly, embryos exposed to H_2_O_2_ exhibited visually delayed epiboly progression at 6 hpf compared to the control group, while NAC and PA-extract groups showed comparable progression to controls, suggesting a protective effect against oxidative stress. Following epiboly measurement at 6 hpf, yolk area was assessed at 8 and 120 hpf to detect developmental delays. No significant variation in yolk area was detected at 8 hpf across all groups (*p* = 0.2862). In contrast, a significant difference emerged at 120 hpf (*p* = 0.001), with the H_2_O_2_ group exhibiting a larger yolk area compared to the control group (82.00 ± 5.292 vs. 78.33 ± 5.686 mm^2^), as confirmed by post hoc Tukey’s test (*p* = 0.0309). Furthermore, all concentrations of PA extract significantly reduced yolk area relative to the H_2_O_2_ group (12.5 ppm, *p*-value = 0.0354; 25 ppm, *p*-value= 0.0029; 50 ppm, *p*-value = 0.0006; Figure 1D), whereas NAC treatment did not result in a statistically significant difference (*p* = 0.1304).

Furthermore, the results revealed that exposure to H_2_O_2_ reduced hatching rate, and that recovery with NAC and PA treatment was dose-dependent (Figure 1A). H_2_O_2_ exposure inhibited embryo hatching by approximately 30%. Notably, at the highest extract concentration (50 ppm), the hatching rate reached 100%, comparable to the control group. A comparable pattern was observed for body length, which was significantly reduced following H_2_O_2_ exposure (Figure 1C). Greater significance was observed at longer observation time points, with *p*-value of 0.0003 at 72 hpf and a *p*-value of 0.0001 at 96 and 120 hpf, which suggests the effects of treatment on zebrafish development are time-dependent. Compared with the H_2_O_2_ group, NAC did not result in a significant recovery (*p* = 0.8284 at 72 hpf; *p* = 0.9976 at 96 hpf; *p* = 0.5439 at 120 hpf). Furthermore, the heart rate of developing larvae was further assessed in relation to duration and PA extract concentration. The mean heart rate (beats/10 s) for the control group was 22.67 ± 0.577 at 72 hpf and 28.00 ± 1.000 at 96 hpf, indicating an increased heart rate pattern during development [46]. In contrast, H_2_O_2_-exposed larvae exhibited a significantly higher heart rate than the control group at 96 hpf (*p* < 0.0001), indicating tachycardic effects following oxidative stress (Figure 1B). Consistent with other parameters, treatment with NAC and PA extract restored heart rate toward control levels, with the 50 ppm PA group showing the greatest recovery effect compared with the H_2_O_2_ group.

### 3.3. H_2_O_2_-Induced Behavioral Changes in Zebrafish Larvae

**Figure 2:**
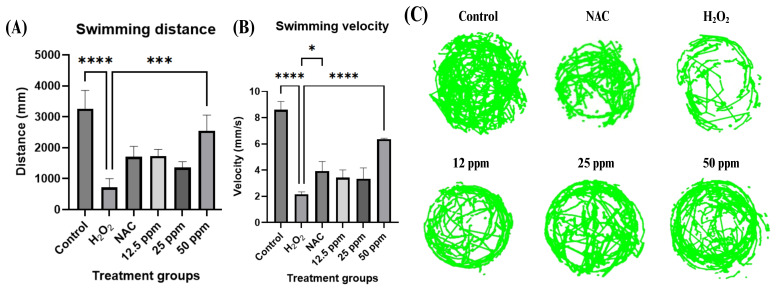
Results of the behavioral changes measurement. Swimming behavior was recorded from 12 larvae per treatment group (*n* = 12). Parameters include (**A**) Swimming distance; (**B**) Swimming velocity; and (**C**) Swimming pattern. (* = *p*-value < 0.05; ; *** = *p*-value < 0.001; **** = *p*-value < 0.0001).

Exposure to H_2_O_2_ markedly impaired locomotor activity, as evidenced by a significant reduction in both swimming distance (721 ± 280.6 mm vs. 3264 ± 586.6 mm in control, *p* < 0.0001) and velocity (2.170 ± 0.1746 mm/s vs. 8.604 ± 0.6344 mm/s in control, *p* < 0.0001) (Figure 2A,B). These findings confirm the successful induction of oxidative stress-mediated behavioral deficits.

NAC treatment resulted in a significant improvement in swimming velocity relative to the H_2_O_2_ group (3.921 ± 0.7424 mm/s vs. 2.170 ± 0.1746 mm/s, *p* = 0.0286), whereas no significant change was observed in swimming distance (1704.86 ± 339.47 mm vs. 754.04 ± 244.47 mm, *p* = 0.0723). Qualitative trajectory analysis further substantiated these findings (Figure 2C). The difference in swimming pattern was most prominent in the H_2_O_2_ group, in which zebrafish larvae exhibited a preference for the well periphery area while avoiding the central open zone. This tendency, known as thigmotaxis, is characterized by preferential movement along the periphery and avoidance of open areas, and is commonly employed as an indicator of anxiety-like behavior in zebrafish [47].

In contrast, larvae treated with NAC and PA extract displayed increased exploration of the central zone when compared to the H_2_O_2_ group. At higher concentrations (25 and 50 ppm), PA extract treatment resulted in increased central zone occupancy and exploratory activity patterns most similar to the control, suggesting a dose-dependent recovery that aligns with the previously observed developmental outcomes.

### 3.4. Nitrite Levels Measured by Griess Assay

**Figure 3:**
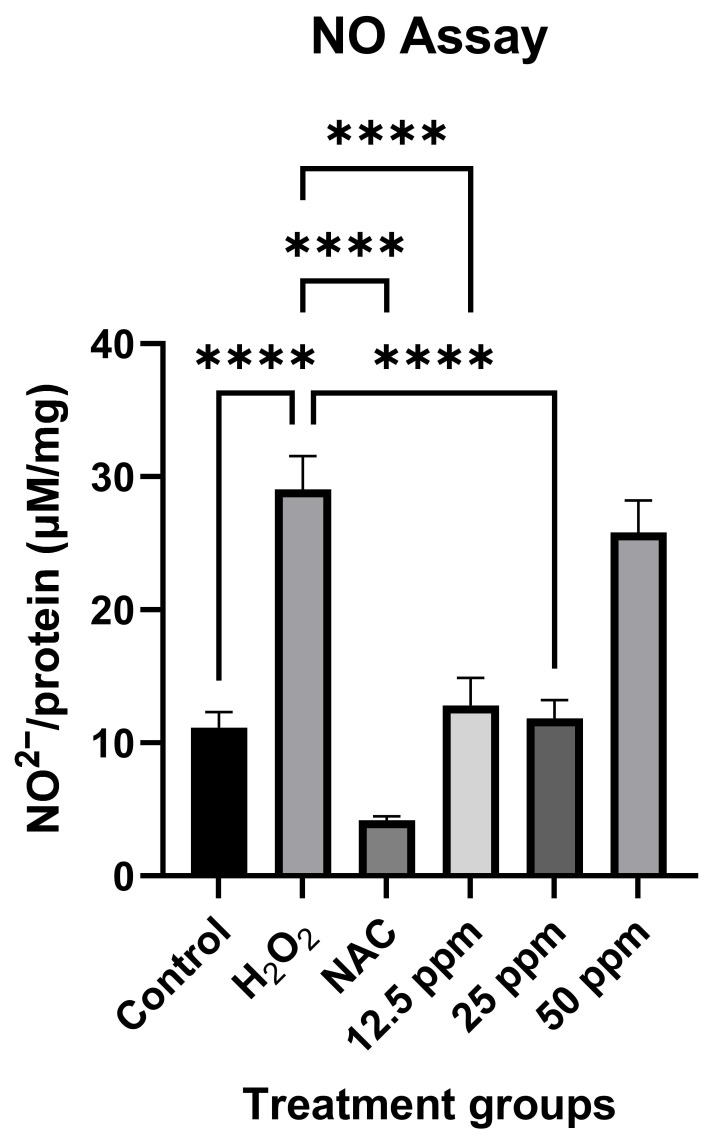
NO^2−^ concentration in zebrafish following treatments (**** = *p*-value < 0.0001).

In addition to the developmental and behavioural findings, nitrite levels were quantified as a marker of nitrosative stress and downstream inflammatory response to H_2_O_2_-induced oxidative challenge. While elevated NO may reflect iNOS induction rather than a direct global increase in ROS, nitrosative stress and oxidative stress are mechanistically interrelated components of H_2_O_2_-induced toxicity. As shown in Figure 3, exposure to H_2_O_2_ significantly increased nitrite levels compared to the control group, with measured NO^2−^ levels of 29.04 ± 2.495 µM/mg and 11.13 ± 1.191 µM/mg, respectively (*p* < 0.0001). Treatments with NAC were notably effective in reducing NO^2−^ levels to 4.19 ± 0.3 µM/mg, lower than in the control group. The PA extract-treated group exhibited a marked reduction in nitrite levels at concentrations of 12.5 ppm and 25 ppm, showing values of 12.82 ± 2.065 µM/mg and 11.85 ± 1.378 µM/mg, respectively. However, treatment with 50 ppm of PA extract did not result in a significant alteration in NO^2−^ levels, which remained at 25.83 ± 2.393 µM/mg.

### 3.5. Metabolomic and Pathway-Level Changes Associated with Treatment

**Figure 4:**
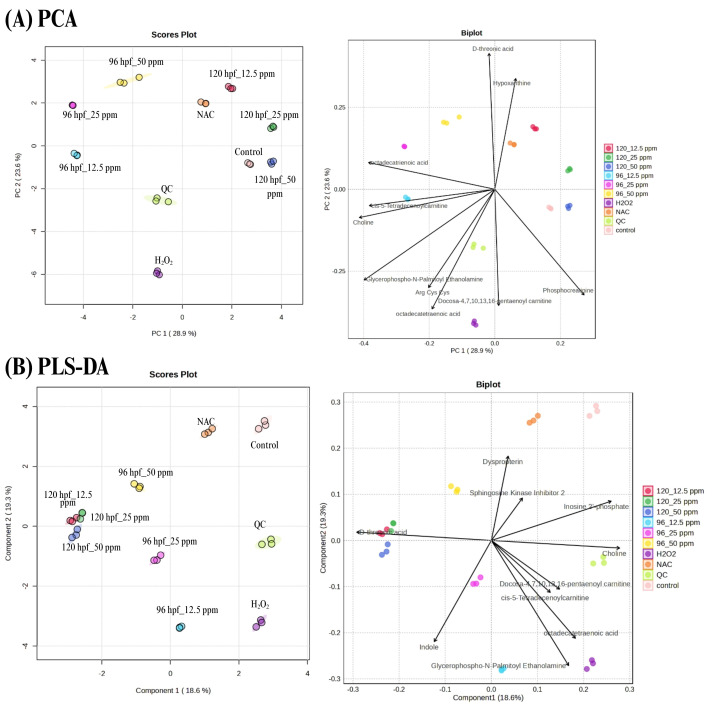
Metabolite profile of zebrafish following treatments, assessed by (**A**) PCA and (**B**) PLS-DA. In biplots, arrow length indicates the magnitude of each metabolite’s contribution to the total variance, and arrow direction reflects the trend of increasing abundance along the axes. QC: quality control; NAC: N-acetylcysteine (positive control); H_2_O_2_ (oxidative stress inducer). Numeric labels denote concentration (ppm) and exposure duration (hpf).

A PCA score plot (Figure 4A) summarizes the overall variation in metabolomic profiles of zebrafish samples. The corresponding biplot illustrates the relationship between treatment groups and annotated metabolites. The length and direction of arrows represent how strongly and toward which group or cluster each metabolite contributed. The first two principal components (PC1 and PC2) together explained approximately 52.5% of the total variance, indicating moderate differentiation among the treatment groups. Distinct separation was observed between the H_2_O_2_ group and the control group. Meanwhile, the metabolic profiles of zebrafish treated with NAC clustered closely with the control samples, suggesting a metabolomic profile similar to that of the non-stressed group. The PA-treated group at 120 hpf clustered closely with the control group, with the 50 ppm concentration showing the closest proximity. A notable pattern was that the 96_50 and 120_12.5 clusters were in close proximity, suggesting similar overall metabolomic states despite different exposure conditions. This observation showed that exposure at 96 hpf to a higher concentration of extract produced a metabolic response comparable to that observed at 120 hpf with a lower concentration.

To further validate and refine the group differences observed in the PCA, a PLS-DA model was constructed (Figure 4B). Unlike PCA, PLS-DA is a supervised method that incorporates group classification to maximize inter-class discrimination. This approach enables clearer visualization of metabolites most responsible for group separation [48]. Compared with PCA, the PLS-DA model showed clearer group separation, confirming that the treatments induced distinct metabolic changes. None of the PA-treated group clusters overlapped completely, indicating that each concentration and incubation period produced a distinct metabolomic pattern. This separation confirms that the PA extract exerted dose-dependent and time-dependent effects on embryo metabolism.

**Figure 5:**
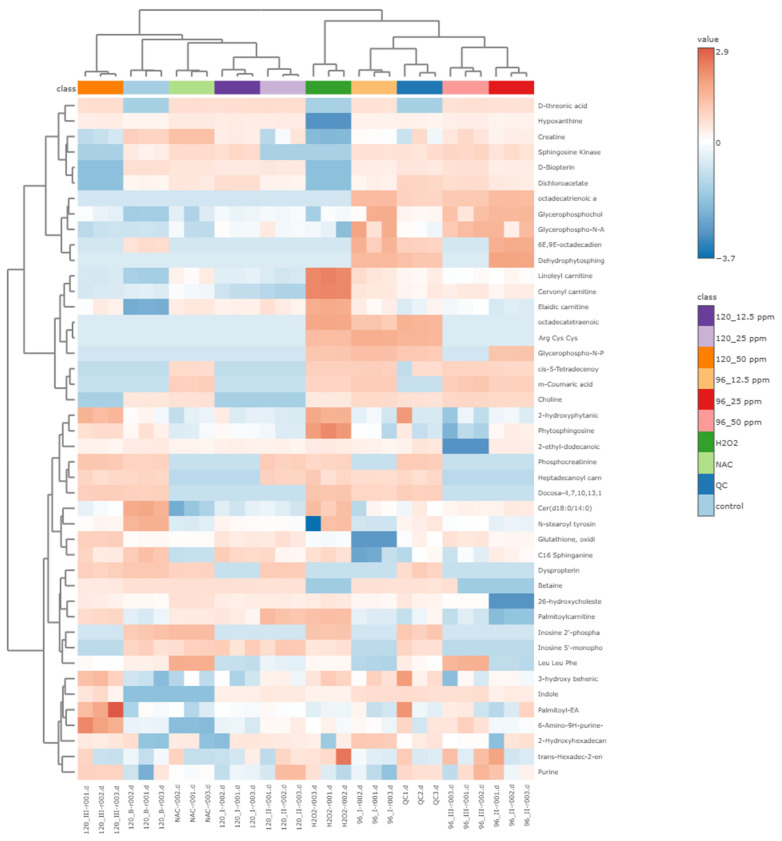
Hierarchical clustering heatmap based on metabolite abundance across treatment groups. Metabolites and samples were hierarchically clustered based on normalized intensity values, with color gradients indicating relative abundance. Red represents higher abundance, and blue represents lower abundance.

The relative abundance of annotated metabolites across all treatment groups is displayed by the hierarchical cluster heatmap (Figure 5). Distinct clustering patterns were observed, with the H_2_O_2_ group forming a separate branch from the control, NAC, and 120 hpf PA-treated groups, indicating marked metabolic differences under oxidative stress. The control, NAC, and 50 ppm PA-treated up to 120 hpf group samples clustered closely together, confirming that NAC treatment restored the metabolic profile toward the non-stressed state. The hierarchical cluster heatmap clearly illustrates that both concentration and exposure time influenced the metabolic response to extract. As the concentration of extract increased (from 12.5 to 50 at 96 hpf), there was a gradual restoration of key metabolites, including C16 sphinganine, dehydrophytosphingosine, and purine, indicating a dose-dependent recovery. When comparing exposure time, embryos treated for 120 min showed a further shift toward the control pattern even at lower concentrations.

**Figure 6:**
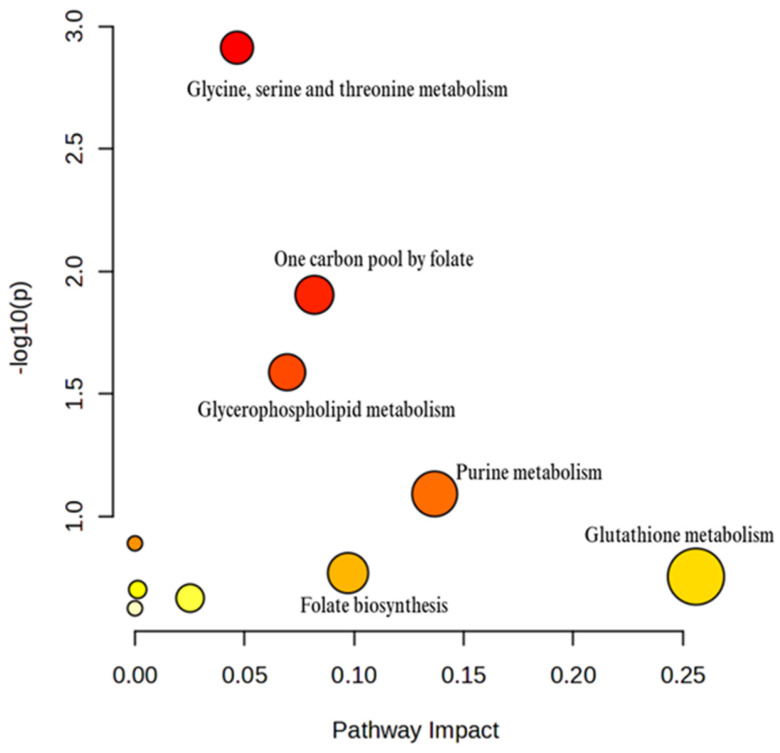
Summary of pathway analysis for significantly altered metabolites following treatments (VIP > 1; *p* < 0.05). Each bubble represents a metabolic pathway with bubble size corresponding to the pathway impact value, where larger bubbles indicate greater pathway influence. Color gradient reflects the degree of statistical significance, where color shifts from red, indicating higher significance, to yellow, indicating comparatively lower significance.

Of the metabolites detected in the spectra, 33 were significantly altered across the treatment groups (*p* < 0.05) and were subsequently selected for pathway analysis (Figure 6, Table 3). Pathway analysis identified multiple metabolic pathways affected by the treatments. Among these, glycine, serine, and threonine metabolism showed the highest statistical significance (*p* = 0.0012), followed by one-carbon via the folate pathway (*p* = 0.0125) and glycerophospholipid metabolism (*p* = 0.0258). Additional pathways, including purine metabolism and glutathione metabolism, were not statistically significant but exhibited the highest pathway impact scores, with values of 0.137 and 0.256, respectively.

## 4. Discussion

The phytochemical profile of *P. angulata* ethanolic leaf extract provides the mechanistic foundation for its observed neuroprotective activity. While the elevated TFC and TPC values confirm an appreciable phenolic content, the antioxidant activity of this extract is not solely attributable to phenolic compounds. Among the annotated LC-MS constituents, physalin A and physalin E, withanolide-class steroidal lactones characteristic of the Solanaceae family [49], have been demonstrated to activate the Nrf2 signaling pathway via ERK and p38 kinase, inducing downstream detoxifying enzymes including NQO-1 and HO-1 [50]. This transcriptionally driven antioxidant mechanism is distinct from and complementary to the hydrogen-donating radical scavenging activity of phenolic compounds such as robinetin 3-rutinoside [51]. It is worth noting that the limited annotation of phenolic acids in the LC-MS data is likely a technical artifact rather than evidence of their absence: the analysis was conducted in positive ESI mode, which has reduced sensitivity for phenolic acids that ionize more efficiently in negative mode, and annotation relied on the METLIN database, which has relatively limited phenolic coverage compared to dedicated phytochemical databases. This is corroborated by the TPC measurement confirming substantial phenolic content in the extract. The phylogenetic proximity of *P. angulata* to *Withania somnifera* further supports the anticipated neuroprotective potential of PA-derived withanolides, given well-characterized activities of the latter in neuronal models [52,53]. However, withanolide safety warrants acknowledgment: prolonged exposure has been associated with hepatotoxic effects, attributed to withanone-mediated DNA adduct formation that may interfere with transcription and repair, a mechanism potentially mitigated by glutathione [22]. Given the phylogenetic similarity between the two species, the safety profile of *P. angulata*-derived withanolides warrants careful evaluation [21]. The concentrations used in this study fall well below the established LC50, and the absence of mortality or morphological abnormalities throughout the treatment period supports the safety of the selected dose range [54].

The selection of 0.5 mM H_2_O_2_ as a sublethal oxidative stressor is well-supported by the literature, which documents observable toxic effects at 0.1–0.5 mM without exceeding 50% mortality, while doses exceeding 2.5 mM can lead to up to 100% mortality within 168 hpf [41,55,56]. This dose induced a robust multi-system oxidative stress phenotype, encompassing developmental toxicity, locomotor impairment, and metabolic disruption, without mortality, confirming its suitability for evaluating neuroprotective interventions. The developmental toxicity observed is mechanistically coherent: reduced hatching rate likely reflects compromised chorion degradation through downregulation of the *cd63* gene [57,58,59]; shortened body length indicates impaired cellular proliferation mediated by pro-apoptotic pathway activation; and increased yolk retention does not reflect energy sufficiency but rather a failure of yolk mobilization, forcing the embryo to catabolize membrane glycerophospholipids as a harmful compensatory energy source [60,61]. This metabolic substitution directly links the developmental toxicity findings to the metabolomic perturbations discussed below. Elevated heart rate further underscores the systemic impact of oxidative stress, likely mediated through CaMKII activation and consequent ion channel disruption [62,63].

The impairment of light-phase locomotor activity and increased thigmotaxis behavior in H_2_O_2_-exposed larvae, despite the absence of gross morphological abnormalities, is consistent with neural rather than structural injury [33,64]. This distinction is important: it positions the observed behavioral deficits as a functional neurotoxicity readout, attributable to reduced AChE activity [65], motor neuron axonal defects [66], and mitochondrial dysfunction impairing synaptic transmission [67,68,69], all of which are consistent with the acylcarnitine accumulation observed in the metabolomic profile, indicating impaired β-oxidation and mitochondrial inefficiency [70,71]. It should be noted that behavioral assessment in this study was confined to light-phase locomotor activity due to equipment constraints. Conclusions regarding behavioral outcomes therefore pertain specifically to light-phase locomotor and anxiety-like behavior, and the full light/dark differential photoresponse could not be characterized. Nevertheless, light-phase swimming distance, velocity, and thigmotaxis are well-validated neurotoxicity endpoints widely employed in published neuroprotection studies [47,64].

PA extract treatment reversed these impairments in a dose- and time-dependent manner, with 50 ppm demonstrating the most consistent recovery across developmental and behavioral endpoints. The time-dependent emergence of significant inter-group differences, minimal at earlier timepoints and pronounced at later developmental stages, reflects the amplification of oxidative stress effects as embryos progress through critical developmental checkpoints [72,73]. This is further consistent with zebrafish growth dynamics, which show the most rapid growth between 60 and 90 days post-fertilization, making later observation windows more sensitive to treatment effects [73]. The superior efficacy of PA extract over NAC is mechanistically attributable to its multi-target phytochemical profile: whereas NAC operates primarily as a glutathione precursor with limited direct ROS reactivity at physiological pH [74], the diverse constituents of PA extract, including Nrf2-activating physalins, membrane-protective flavonoids, and anti-inflammatory chlorophyll derivatives, collectively provide broader mechanistic coverage against H_2_O_2_-induced toxicity [4,10,17,73,74,75,76]. This multi-target action prevented activation of downstream pro-apoptotic pathways and supported metabolic recovery across multiple systems simultaneously [13,77,78].

A particularly informative finding was the dissociation between nitrosative stress levels and functional recovery at 50 ppm PA. While lower PA concentrations (12.5 and 25 ppm) reduced nitrite levels toward control values, treatment at 50 ppm did not suppress NO production, yet achieved the most complete restoration of developmental and behavioral endpoints. This suggests that PA at this concentration confers neuroprotection primarily through restoration of downstream metabolic pathways, including glutathione synthesis, glycerophospholipid homeostasis, and one-carbon pool metabolism (Table 3, Figure 6), rather than through direct suppression of nitrosative signaling. The precise mechanistic basis of this dissociation remains to be fully elucidated. Nitrosative signaling is known to exert dual roles in the CNS, acting as both a neuroprotective and neurotoxic mediator depending on concentration and context [79,80,81], and future studies should clarify its specific role in PA-mediated recovery [82,83].

Metabolomic analysis provides the integrating framework that mechanistically explains the developmental, behavioral, and nitrosative findings. The accumulation of acylcarnitines and PUFAs in H_2_O_2_-exposed larvae directly indicates mitochondrial β-oxidation impairment [70,71], explaining both the energy deficit underlying developmental delay and the synaptic dysfunction driving locomotor impairment. The compensatory mobilization of membrane glycerophospholipids as an alternative energy source [84,85] connects the yolk retention findings to broader membrane disruption and explains the reduced larval mobility observed behaviorally [33,86]. A reduction in glycerophospholipids, along with biplot findings, further suggests that H_2_O_2_ exposure increased the mobilization of fatty acids as an alternative energy source to compensate for oxidative-stress-induced energy deficits, consistent with the impaired yolk mobilization discussed above [87,88]. Disruption of glycine, serine, and threonine metabolism, the most statistically significant dysregulated pathway, carries direct neurodevelopmental implications: L-serine is an NMDA receptor agonist precursor and essential for myelin synthesis; glycine functions as an inhibitory neurotransmitter; and their depletion represents a mechanistic basis for the neural impairment underlying the behavioral deficits observed [89,90]. The concurrent disruption of one-carbon pool metabolism, reflected by reduced choline and betaine, further impairs methionine homeostasis, purine biosynthesis, and glutathione synthesis in an interconnected manner [91,92]. A time-dependent staged metabolic recovery was observed in PA-treated groups, as evidenced by choline restoration at 96 hpf, which supported membrane and neurotransmitter repair [82,83], followed by betaine elevation at 120 hpf, indicative of redox stabilization [91,93]. This sequential metabolic restoration mirrors and mechanistically accounts for the time-dependent biological recovery documented across developmental and behavioral endpoints.

Central to this recovery is the restoration of GSH, whose depletion under oxidative stress represents a critical failure point leading to inhibited growth and neuromotor deficits [33,84,94]. The Nrf2-activating capacity of physalins in the extract [50] directly accounts for this GSH restoration by inducing glutathione synthesis enzymes, thereby replenishing endogenous antioxidant defenses [85]. Complementarily, robinetin 3-rutinoside contributes membrane protection through direct radical scavenging, preventing lipid peroxidation and preserving glycerophospholipid integrity [95]. The detection of dyspropterin in PA-treated groups further suggests maintenance of the BH4 biosynthetic pathway, supporting neurotransmitter precursor synthesis, a pathway commonly disrupted in neurological disorders [96,97], while elevated D-threonic acid reflects enhanced ascorbate turnover as part of the antioxidant response [98,99,100].

Taken together, these converging lines of evidence establish a coherent mechanistic narrative: H_2_O_2_-induced oxidative stress disrupts mitochondrial function, depletes GSH, impairs glycerophospholipid homeostasis, and dysregulates amino acid and one-carbon metabolism, collectively producing the developmental and behavioral deficits observed. PA extract reverses these disruptions through complementary multi-target mechanisms: Nrf2-mediated GSH restoration, membrane lipid protection, amino acid pathway normalization, and neurotransmitter pathway support. The resilience of this protective effect despite sustained NO levels suggests a mechanistic robustness that may confer advantages over single-agent antioxidants in conditions of complex or sustained oxidative stress, a distinction with potential implications for the development of natural product-based neuroprotective strategies. Regarding the metabolomic design, samples were collected at 96 hpf for PA-treated groups and 120 hpf for all groups to capture both acute perturbations and fully matured larval metabolic profiles [41,101]; PCA and PLS-DA comparisons were conducted exclusively within the 120 hpf dataset to prevent developmental stage from confounding treatment-related metabolic discrimination.

Several limitations of this study should be acknowledged in the context of interpreting these findings. First, while zebrafish share conserved neurotransmitter systems and metabolic pathways with mammals, the neuroprotective effects observed have not been validated in mammalian models, and differences in pharmacokinetics, blood–brain barrier structure, and metabolic scaling limit direct translational inference. Second, the H_2_O_2_ immersion model represents an exogenous oxidant challenge that may not fully recapitulate the compartment-specific, source-dependent endogenous ROS generation operative in human neurological conditions. Third, the use of a crude extract precludes definitive attribution of effects to individual bioactive constituents, though it reflects the holistic therapeutic potential of the plant. Fourth, the untargeted metabolomic approach, combined with sampling at two developmental timepoints, means that some metabolic shifts may partially reflect developmental transitions rather than treatment-specific effects. Fifth, behavioral conclusions are confined to light-phase locomotor activity due to equipment constraints, and epiboly progression, assessed qualitatively under stereomicroscopy without quantitative imaging, has been reported as a supplementary developmental observation rather than a primary endpoint, given its occurrence prior to the onset of neurogenesis. Finally, the absence of direct in vivo ROS quantification and the mechanistic basis of the dissociation between persistent nitrosative stress and functional recovery observed at 50 ppm PA represent additional limitations that remain to be fully elucidated. Future studies employing isolated compound testing, mammalian model validation, dual-polarity LC-MS analysis, targeted metabolomics, dedicated infrared behavioral tracking systems, fluorescent ROS probes (e.g., DCFH-DA), iNOS expression analysis, and enzymatic antioxidant markers (SOD, CAT, GPx activity) would substantially strengthen and expand upon the mechanistic and translational conclusions drawn here.

## 5. Conclusions

The present study demonstrates that *P. angulata* leaf extract confers multi-target neuroprotection against H_2_O_2_-induced oxidative stress in zebrafish, with superior efficacy over NAC across multiple developmental and behavioral endpoints. A particularly noteworthy finding was the dissociation between nitrosative stress and functional recovery at 50 ppm: despite persistent NO levels, near-complete restoration of locomotor and developmental parameters was achieved, suggesting PA-mediated neuroprotection operates downstream of nitrosative signaling through restoration of glutathione synthesis, glycerophospholipid homeostasis, and one-carbon pool metabolism. These findings should be interpreted within the context of the study’s limitations, including the absence of mammalian model validation, the use of a crude extract, restriction of behavioral assessment to light-phase locomotor activity, untargeted metabolomic design, the absence of direct in vivo ROS quantification, and the mechanistic basis of the dissociation between persistent nitrosative stress and functional recovery at 50 ppm PA. Future studies addressing these limitations would substantially strengthen the mechanistic and translational conclusions drawn here. Nevertheless, the convergent evidence from developmental, behavioral, and metabolomic analyses establishes *P. angulata* as a compelling candidate for further development as a natural neuroprotective agent.

## Figures and Tables

**Table 1:** Antioxidant capacity assay results.

Sample	DPPH IC_50_ Value (ppm)	ABTS IC_50_ Value (ppm)	FRAP (g FeSO_4_ eq/100 g)
Ascorbic acid	4.567 ± 0.08	2.694 ± 0.05	79.215 ± 0.83
PA extract	114.91± 2.19	66.663 ± 0.72	4.163 ± 0.08

**Table 2:** Compounds identified in *P. angulata* ethanolic extract through LC-MS/QTOF analysis.

No	Compound	[M-H]^+^	*m*/*z*	Measured Mass	Theoretical Mass	Class	Peak Height (cps)
1	2-amino-3-methyl-1-butanol	C_5_H_14_NO	104.1073	103.1	103.0997	Alcohol amines	75,585
2	D-Proline	C_5_H_10_NO_2_	116.0708	115.0633	115.0633	Amino acid	25,700
3	Pheophorbide A	C_35_H_37_N_4_O_5_	593.2761	592.2686	592.2686	Chlorophyll derivatives	17,412
4	25,27-dihydro-4,7-didehydro-7-deoxyphysalin A	C_28_H_31_O_9_	511.1967	510.1893	510.189	Withanolides	15,201
5	N-Cyclohexanecarbonylpentadecylamine	C_22_H_44_NO	338.3423	337.3349	337.3345	Amides (fat derivatives)	14,125
6	Physalin A	C_28_H_31_O_10_	527.1912	526.1882	526.1839	Withanolides	13,170
7	4-methylaminobutyrate	C_5_H_12_NO_2_	118.0865	117.0783	117.079	Amino acid derivatives	11,336
8	N-(1-Deoxy-1-fructosyl)isoleucine	C_12_H_24_NO_7_	294.1543	293.147	293.1475	Amino acid derivatives	11,169
9	N-(1-Deoxy-1-fructosyl)valine	C_11_H_22_NO_7_	280.1399	279.1326	279.1318	Amino acid derivatives	8993
10	Emmotin A	C_16_H_23_O_4_	279.1597	278.1524	278.1518	Tetralins	7735
11	N6-Methyl-2′-deoxyadenosine	C_11_H_16_N_5_O_3_	266.124	265.1166	265.1175	Nucleosides	6349
12	Robinetin 3-rutinoside	C_27_H_31_O_16_	611.1608	610.1536	610.1534	Glycoside flavonoids	5646
13	9Z,12Z,15E-octadecatrienoic acid	C_17_H_31_O_2_	279.2323	278.2254	278.2246	Unsaturated fatty acids	5231
14	Physalin E	C_28_H_33_O_11_	545.2016	544.1945	544.1945	Withanolides	5062

**Table 3:** Pathway analysis results.

Pathway	*p* Value	−Log(*p*)	Impact
Glycine, serine, and threonine metabolism	0.0012227	2.9127	0.04655
One carbon pool by folate	0.01246	1.9045	0.08187
Glycerophospholipid metabolism	0.025797	1.5884	0.06944
Purine metabolism	0.080833	1.0924	0.13674
Folate biosynthesis	0.17001	0.76953	0.09722
Glutathione metabolism	0.17577	0.75505	0.25596
Sphingolipid metabolism	0.19847	0.70230	0.00125
Arginine and proline metabolism	0.21512	0.66731	0.02516

## Data Availability

The datasets presented in this article are not readily available because access is restricted due to their size and complexity, and in accordance with institutional data management policies. Requests to access the datasets should be directed to the corresponding author.

## Supplementary Materials

The following supporting information can be downloaded at: https://www.mdpi.com/article/10.3390/antiox15060758/s1, Figure S1: Dried leaf extraction procedure; Figure S2: Zebrafish embryo treatment procedures; Figure S3: Metabolomic analysis sample extraction and reconstruction workflow; Figure S4: Chromatogram and mass spectra of the *P. angulata* ethanolic extract obtained by LC-MS/QTOF in ESI positive mode; Figure S5: Representative images of epiboly progression; Table S1: Total flavonoid, phenolic content, and yield of the *P. angulata* extract.

## Abbreviations

The following abbreviations are used in this manuscript:

AChE	Acetylcholinesterase
ADHD	Attention Deficit Hyperactivity Disorder
AlCl_3_	Aluminum chloride
ANOVA	Analysis of Variance
ASD	Autism Spectrum Disorder
BBB	Blood–Brain Barrier
BH_4_	Tetrahydrobiopterin
CaMKII	Calmodulin-dependent protein kinase II
CNS	Central Nervous System
dpf	Days Post-Fertilization
GPC	Glycerophosphocholine
GSH	Glutathione
H_2_O_2_	Hydrogen peroxide
HMDB	Human Metabolome Database
hpf	Hours post-fertilization
LC_50_	Lethal Concentration 50%
LC–MS	Liquid chromatography–mass spectrometry
NAC	N-acetylcysteine
NMDA	N-methyl-D-aspartate
OECD	Organisation for Economic Co-operation and Development
PA	*Physalis angulata* L.
PCA	Principal component analysis
PC	Principal component
PE	Phosphatidylethanolamine
PEA	Palmitoylethanolamide
PLS-DA	Partial least squares–discriminant analysis
PUFAs	Polyunsaturated fatty acids
QC	Quality control
QE	Quercetin equivalent
ROS	Reactive oxygen species
rpm	Revolutions per minute
RSD	Relative standard deviation
SD	Standard deviation
TFC	Total flavonoid content
VIP	Variable importance in projection
