# Supplementary material for: Physalis angulata Leaf Extract Attenuates H2O2-Induced Neurotoxicity in Zebrafish Through Metabolomic Evidence of Antioxidant Pathway Restoration
Source: Antioxidants (Basel). 2026 Jun 16;15(6):758. doi: 10.3390/antiox15060758 (PMC13295755; doi:10.3390/antiox15060758)
Supplement: Supplementary file 1 [file antioxidants-15-00758-s001.zip › antioxidants-4311666-supplementary.pdf]

Supplementary Materials

Physalis angulata Leaf Extract Attenuates H_2O_2-Induced Neurotoxicity in Zebrafish Through Metabolomic Evidence of Antioxidant Pathway Restoration

Table S1. Total flavonoid, phenolic content, and yield of the *P. angulata* extract

Total Flavonoid Content (mg QE/g Extract)	Total Phenolic Content (mg GAE/g Extract)	Yield (%)
51.06 ± 0.34	59.01 ± 0.79	15.64%

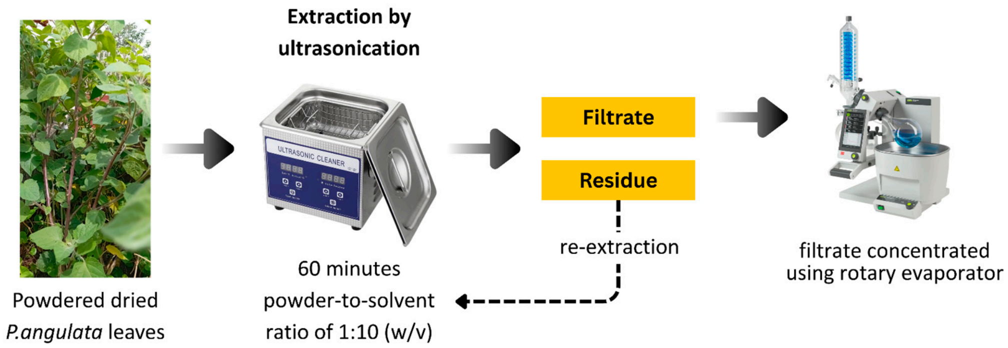

Figure S1. Dried leaves extraction procedure

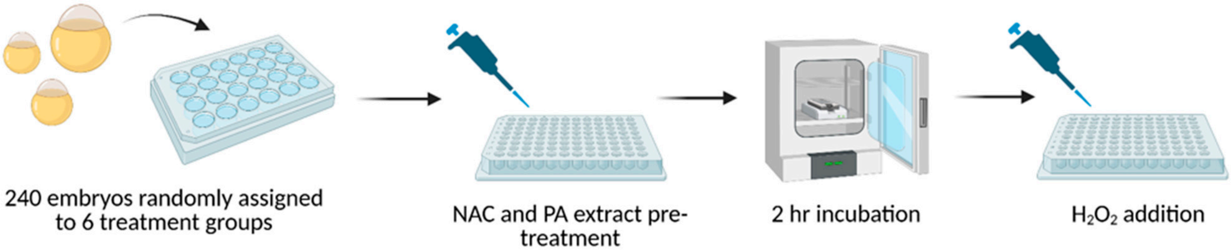

Figure S2. Zebrafish embryos treatment procedure. Created in BioRender. Maretta, D. (2026) https://BioRender.com/if3o5mu

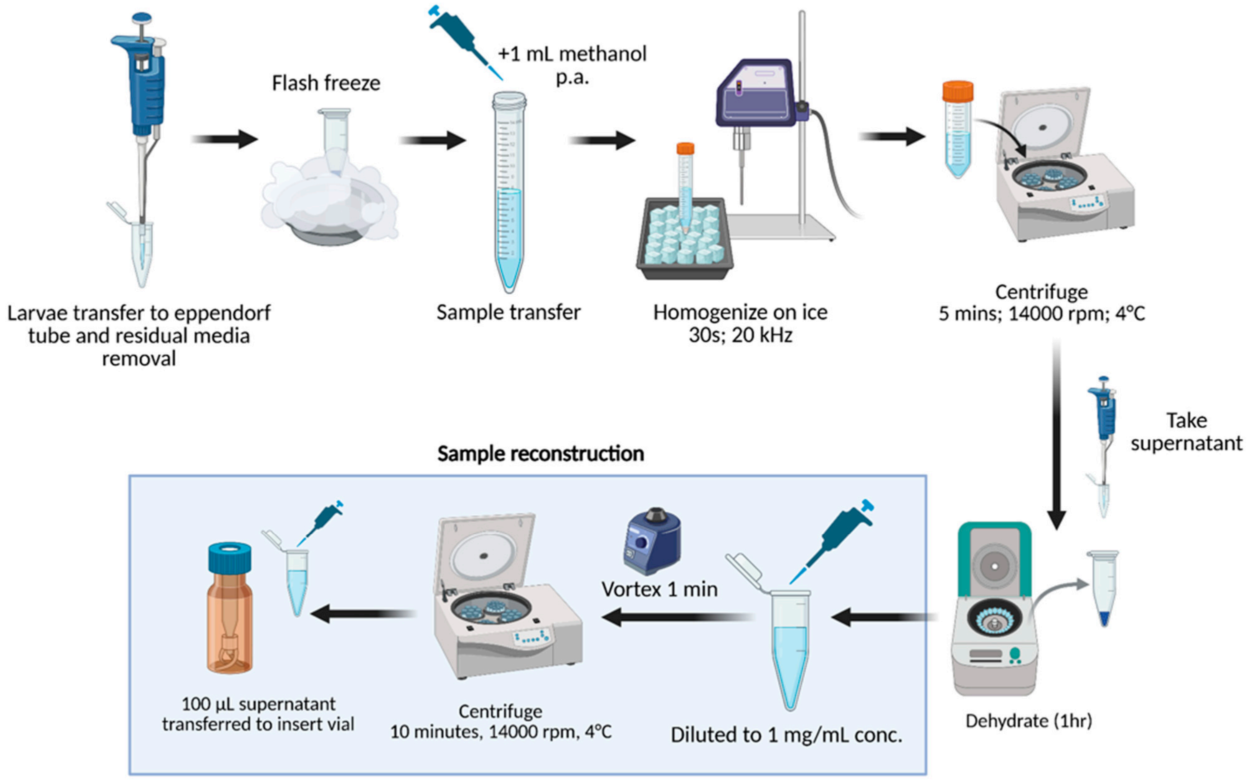

Figure S3. Metabolomic analysis sample extraction and reconstruction workflow. Created in BioRender. Maretta, D. (2026) https://BioRender.com/2vey4l8

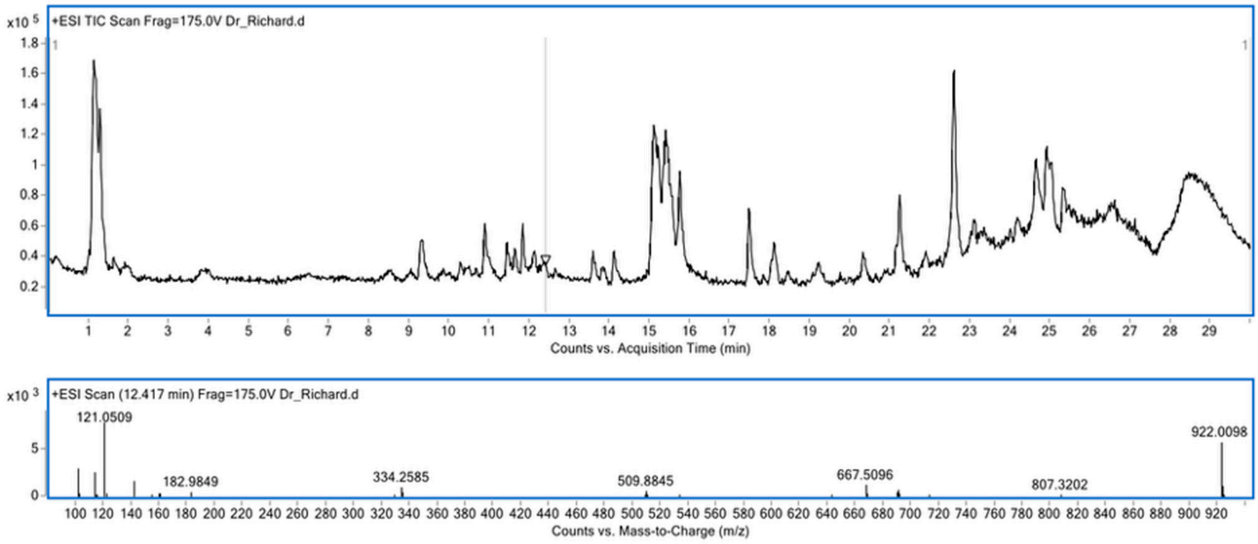

Figure S4. Chromatogram and mass spectra of the *P. angulata* ethanolic extract obtained by LC-MS/QTOF in ESI positive mode.

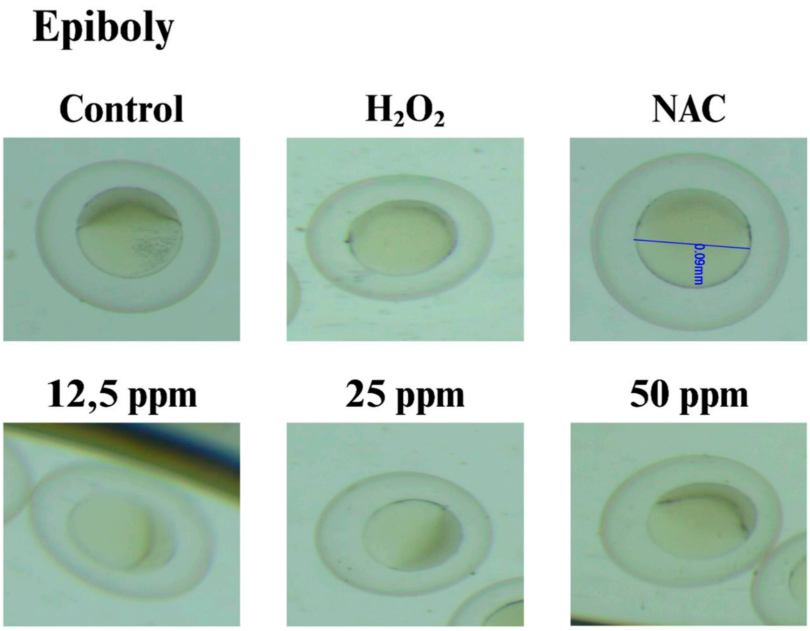

Figure S5. Epiboly progression was qualitatively observed under stereomicroscopy at 6 hpf.
